# A large-scale empirical investigation of specialization in criminal career

**DOI:** 10.1038/s41598-023-43552-6

**Published:** 2023-10-11

**Authors:** Georg Heiler, Tuan Pham, Jan Korbel, Johannes Wachs, Stefan Thurner

**Affiliations:** 1 Complexity Science Hub Vienna, Vienna, Austria; 2 Technical University Vienna, Vienna, Austria; 3 Medical University of Vienna, Vienna, Austria; 4 Corvinus University of Budapest, Budapest, Hungary; 5 Centre for Economic and Regional Studies, Budapest, Hungary; 6 Santa Fe Institute, Santa Fe, USA

**Keywords:** Scientific data, Complex networks, Human behaviour

## Abstract

We use a comprehensive longitudinal dataset on criminal acts over 6 years in a European country to study specialization in criminal careers. We present a method to cluster crime categories by their relative co-occurrence within criminal careers, deriving a natural, data-based taxonomy of criminal specialization. Defining specialists as active criminals who stay within one category of offending behavior, we study their socio-demographic attributes, geographic range, and positions in their collaboration networks relative to their generalist counterparts. Compared to generalists, specialists tend to be older, are more likely to be women, operate within a smaller geographic range, and collaborate in smaller, more tightly-knit local networks. We observe that specialists are more intensely embedded in criminal networks, suggesting a potential source of self-reinforcing dynamics in criminal careers.

## Introduction

Researchers of criminal careers aim to understand how past criminal behavior predicts or relates to possible future offending^1,2^. In this context, a subpopulation of criminals of particular interest are re-offenders, those who repeatedly break the law. Criminologists studying re-offenders continue to debate the extent and degree of their specialization, an individual’s repeated commission of similar kinds of crime^3^. If many re-offenders specialize as bank robbers, computer hackers, drug dealers, or burglars, tailored policies for such subpopulations in policing and rehabilitation may be effective^4^, and criminal behavior may be more predictable^5^. If re-offenders tend to remain generalists and exhibit versatility, however, such policies may be ineffective.

Several theoretical frameworks of criminal behavior offer different insights into why re-offenders may specialize or remain versatile^2^. For example, the theory of heterogeneity suggests that it is rather a person’s time-invariant characteristics like their temperament or level of self-control that predicts repeat-offending^6,7^. In other words, differences (i.e., heterogeneities) in innate traits or attitudes of people are the decisive factors in offending outcomes. Heterogeneity suggests that re-offenders will likely be generalists, manifesting specialization only when external opportunities and incentives are consistent.

On the other hand, state dependence theory states that life events and social context can significantly affect inherent propensities to offend and re-offend^8^. For instance, contact with other offenders in prison may introduce new criminal opportunities or result in gang membership^9^, which increases the risk of re-offending. Even the social context of official interactions between offenders and law enforcement, for instance, via parole officers, is thought to shape future behavior^10^. State dependence theory implies that policy interventions, for example, job programs for recently released criminals^11^, can reduce recidivism. At the same time, state dependence suggests that re-offenders will often specialize via processes like social contagion^12,13^ or learning^14,15^. Perhaps synthesizing the two perspectives of heterogeneity and state dependence, Wikstrom’s Situational Action Theory (SAT) emphasizes the dynamic nature of criminal behavior as a function of both individual propensities and changing environmental factors^16^.

These and other theoretical frameworks, including the general^6^ versus typological^17,18^ debate, tend to suggest that either generalist or specialist behavior should be more frequently observed patterns of criminal careers^2^. At the same time, specialization and its conceptual framing are not merely of theoretical interest. Whether an individual is a (potential) generalist or specialist has implications for their potential rehabilitation^19^. Specialization also seems to play an important role in the context of organized crime^20–22^ and in gangs^23^, where it facilitates an efficient division of labor. In this context, specialized individuals may play a key role in the functioning of the larger group^24,25^.

In practice, both specialized and generalist individuals are observed in various empirical studies^18,26^. One survey of the literature from 2003 notes, however, that generalist patterns of offending are more common^1^. That said, variations between study designs, data sources, scope, scale, and measurement make comparisons between studies difficult^27^. Indeed, despite the importance of potential specialization in criminal careers, large-scale empirical evidence is limited, likely owing to the challenges of procuring and handling relevant data. Exceptions include longitudinal studies of cohorts, for example, Andersson et al., who track the criminal careers of a group of juvenile offenders up to age 30^28^ and find gender differences in behavior. Falk et al. study the distribution of violent crime convictions across the Swedish population over a period of decades, finding the intense concentration of activity in a small sub-population^29^. However, both of these works rather study whether it is possible to identify coherent trajectories in criminal careers in terms of their persistence and how they relate to ages^30^, rather than specialization as we frame it. In general, such studies tend to focus on specific kinds of crimes (violent crimes) or age groups (i.e., juveniles^18,31^).

Hence, despite the importance of specialization in criminal careers for policy and associated theoretical debates, there is a clear need for additional empirical measurements of the phenomenon. This article aims to provide an empirically rigorous accounting of the prevalence of specialization in a large population of offenders and to observe differences in socio-demographic attributes, geographic mobility, and collaboration network positions of specialists and generalists. We use a complete dataset of criminal *charges* against individuals in a small central European country over six years. Specifically, our data includes over 1.2 million of distinct criminal events and nearly 600 thousand individuals charged.

To quantify specialization among this population, we refine an information-theoretic method of Tumminello et al.^32^ to cluster types of criminal events that frequently co-occur within individual careers. Our extension of the method considers how often individuals commit different kinds of crime. We define specialists as re-offenders with all their criminal charges within one cluster. A by-product of our method is a measure of the tendency of specialization within different types of criminal activity. For example, individuals do not often specialize in violent crimes^33^ but do specialize in other areas, such as sexual crimes and computer criminality.

We use this classification to both revisit previously studied associations between specialization and socio-demographic attributes like age and gender and to observe new associations between specialization and complex characteristics of criminal behavior. We also study differences between the collaboration network positions of two populations. Differences in the characteristics of specialists and generalists may manifest in significant differences in how they actually cooperate with other criminals. After all, if state-dependent mechanisms such as social learning explain specialization tendencies, it is likely that we can observe differences in how specialists and generalists collaborate. The relative positions of specialists and generalists in the network of criminal collaboration can suggest how human capital (i.e. specialization) and social capital (i.e. important network positions) coordinate in criminality^25,34^.

## Specialization in criminal careers

An important step in defining the specialization of criminal behavior is to group different crime types according to a reasonable taxonomy. Assault and battery, for example, are crimes with legal definitions. To the layman, they are clearly related: no one would be surprised if a criminal was convicted of both offenses over the course of a career. Computer hacking and grave robbing, on the other hand, are intuitively less likely to be carried out by the same person. While legal codes tend to group different kinds of crimes into reasonable categories, such categorizations are not always useful for describing criminal specialization because they may reflect artifacts of the historical evolution of the law^35^. The structure of criminal codes also differs significantly between countries and even within countries over time^36,37^. For instance, crimes committed with a computer are sometimes grouped into their own category, other times linked to their nearest offline counterparts (i.e. fraud)^38^.

The inadequacy of using the legal code to categorize behavior for the purpose of studying specialization is further evidenced by the existence of widely referenced “Crime Classification Manuals” which provide additional categorizations of types of crimes^39^, and international efforts to harmonize categorizations of criminal activity such as The International Classification of Crime for Statistical Purposes (ICCS)^40^. As the aim of these efforts is not to define similarities between criminal acts in terms of their co-occurrence in criminal careers, they are in some ways unsuited to defining specialization. Indeed, we will see that our data-driven clustering of criminal activity does not conform to categorizations in the legal code.

We, therefore, adopt a statistical approach to group crimes that are carried out by the same criminals within individual careers. The goal of this method is to facilitate the categorization of individuals as specialists or generalists using data. We compare the observed distributions of co-occurrence of crimes in the overall population against a statistical benchmark derived from a null model. This null model assumes a randomized distribution of criminal activity across criminals. The grouping of statistically significant co-occurrences of different crime categories within careers creates a self-generated data-driven typology of crime types. Our approach extends methods developed by Tumminello et al.^32^ by considering not only the number of crimes but also the number of perpetrators committing any two types of crimes.

More explicitly, our method to define specialists takes the following steps. First, we define a co-occurrence network of crimes, defined by the legal code, within individual criminal careers. In this network, two crimes, for instance, assault and computer hacking, are connected by a weighted edge counting the frequency that any one person is charged with those two crimes. This network is quite dense and needs to be filtered; in other words, the edges need to be statistically validated. With the resulting statistically validated network of crimes, we are able to detect which kinds of crimes often co-appear in the same careers across the entire population. Next, we apply a clustering algorithm, grouping the nodes (corresponding to crimes defined by the legal code) into clusters of crime categories. For instance, one such category includes the crimes of fraud, embezzlement, and forgery; another includes rape and sexual harassment. Finally, we use this categorization to define criminals as specialists or generalists: a criminal is a specialist if they only commit crimes from a single category across their observed career, otherwise, we say they are generalists. We then carry out an analysis of how these two types of criminals differ, i.e. in terms of their socio-demographic features like age and gender, their geographic mobility, or in how they collaborate with other criminals.

### Data and networks

For this work, we used the anonymized dataset of criminal police statistical data. We gained access to the anonymized database as part of a joint research project with the project partner (see the data availability statement). The dataset contains any criminal charges brought by the police against individuals in the country from 2015-01-01 until 2021-11-09. In total, there were over 580k perpetrators charged in 1.2 million distinct crimes. These events take place in the focal country, with only a small fraction of the events (0.47%) taking place abroad. Due to data privacy reasons, our data cannot be shared publicly.

For each event (criminal act), we observe:

- Location (political region), *r*
- Tme of the act (date), *t*
- Category (legal paragraph of the act), *c*
- Demographics (age and gender at the time of arrest), ${X}_{p}^{\textrm{age}}$, ${X}_{p}^{\textrm{sex}}$
- Unique, anonymized, and persistent identifier of the perpetrator, *p*
- Unique identifier of the criminal act *M*

We note that one criminal activity happening in a particular location at a given time may involve several perpetrators, and may be composed of several crime categories. For example, think of a robbery of a house that involved five criminals who committed the crimes of robbery and murder.

We use this data to define a bipartite network connecting crime types with perpetrators. In particular, we define a rectangular matrix with two indices, $M_{cp}$ with entries counting the number of crimes of type *c* committed by perpetrator *p*, aggregated over time *t*, and regions *r*. The matrix $M_{cp}$ is a bipartite and weighted network. For the binary (unweighted) adjacency matrix ignoring counts, we write $A_{cp}:= \min \{M_{cp},1\}$. From this bipartite network, we will derive two monopartite networks: the crime-crime transition network and a criminal collaboration network.

### Crime-crime transition network

We first project the bipartite network onto the crime categories. The resulting network consists of crime categories as nodes, which are connected by a link if there is a criminal who is charged with both categories. The edges are weighted, increasing in the frequency with which categories co-appear in criminal careers. Mathematically, starting from the bipartite network viewed as a matrix, $M_{cp}$, the projection yields a directed crime-to-crime (C-C) network, where $\textbf{N}_{cd} = \sum _{p} A_{cp}A_{dp}$ corresponds to the number of perpetrators who committed both crimes *c* and *d*.

This network is quite dense because of the scale of our data: there are rare examples of category co-occurrences within specific careers. We, therefore, extract a sparser statistically validated network by filtering the dense, weighted crime co-occurrence network using the method of *hypergeometric filtering*^41^. We define $\textbf{N}_{a} = \sum _b \textbf{N}_{ab}$ as the number of perpetrators who committed crime *a*, and $\textbf{N} = \sum _a \textbf{N}_a$ is the total number of perpetrators. To determine whether the link between crime categories *a* and *b* is significant, we define its corresponding *p*-value as

1 $$\begin{aligned} p_{val}(\textbf{N}_{ab}) = 1 - \sum _{x=0}^{\textbf{N}_{ab}-1} \frac{{\textbf{N}_a \atopwithdelims ()x} {{\textbf{N}-\textbf{N}_a} \atopwithdelims (){\textbf{N}_b -x}} }{{\textbf{N} \atopwithdelims ()\textbf{N}_b}} \,, \end{aligned}$$

which is the cumulative density function of the hypergeometric distribution at ${\textbf {N}}_{ab}-1$. It is the probability that out of $\textbf{N}_a$ and $\textbf{N}_b$ perpetrators who committed crimes of types *a* or *b*, respectively, there is less than ${\textbf {N}}_{ab}$ perpetrators who committed both crimes of type *a* and *b*. A link is considered significant at the significance level, *p*, if $p_{val}(a,b) < p$.

Due to multiple hypothesis testing, we introduce a Bonferroni correction^42^, where an adjusted *p*-value, *p*/*m*, is used, where *m* is the number of tested hypotheses, which –in our case– is the number of links of the C-C network. We use this simplest and quite conservative approach also because it has been shown that more sophisticated corrections, such as the Šidák correction^43^, the Bonferroni-Holm method^44^, or the false discovery rate^45^, all yield similar community structures^32,41^. The resulting network is called *statistically validated network*, and we denote it by $\mathscr {N}_{ab}$.

By following the approach of Tuminello et al.^41^, we are ignoring some important aspects of the data. In particular, we lose information contained in the simplified matrix, $M_{cp}$, by considering only the unweighted matrix, $A_{cp}$ when constructing the C-C projection. Relevant information is lost, such as the number of crimes of a given type, say *b*, that were committed by perpetrators committing two types of crimes, say *a* and *b*. To overcome this we extend the approach by Tuminello et al. We define $\mathscr {P}(a,b)$, as the set of perpetrators who committed crimes of both categories *a* and *b*. The projection of the bipartite network is therefore defined as $\textbf{M}_{ab} = \sum _{p \in \mathscr {P}(a,b)} M_{pb}$, which is a number of crimes of type *b* committed by perpetrators who committed both *a* and *b*. Note that here the matrix $\textbf{M}$ cannot be expressed in terms of matrix multiplication of matrix *M*. The resulting C-C network is directed since, in general, $\textbf{M}_{ab} \ne \textbf{M}_{ba}$.

Again, we exclude links that are not statistically significant. Note that links do not have to be significant in both directions. Therefore we validate if the number of crimes $\textbf{M}_{ab}$ committed by $\textbf{N}_{ab}$ perpetrators is statistically significant. To this end, we define $\textbf{M}_b = \sum _a \textbf{M}_{ab}$ as the number of crimes of type *b*. We consider a random distribution of $\textbf{M}_b$ crimes to $\textbf{N}_b$ perpetrators, where each perpetrator committed at least one crime. Simple combinatorics yields the number of such divisions as ${\textbf{M}_b-1} \atopwithdelims (){\textbf{N}_b-1}$. The crimes can be divided into two groups of perpetrators, the ones who also committed crime *a* and those who did not. Since the number of crimes committed by perpetrators who also committed crime *a* is $\textbf{M}_{ab}$, the total probability that $\textbf{N}_{ab}$ perpetrators out of $\textbf{N}_b$ who committed $\textbf{M}_b$ crimes would commit $\textbf{M}_{ab}$ crimes can be expressed as

2 $$\begin{aligned} p(\textbf{M}_{ab}|\textbf{N}_{ab},\textbf{M}_b,\textbf{N}_b) = \frac{{{\textbf{M}_{ab}-1} \atopwithdelims (){\textbf{N}_{ab}-1}} {{\textbf{M}_b - \textbf{M}_{ab}-1} \atopwithdelims (){\textbf{N}_b - \textbf{N}_{ab}-1}}}{{{\textbf{M}_b-1} \atopwithdelims (){\textbf{N}_b-1}}} \,. \end{aligned}$$

Thus, the *p*-value corresponding to $\textbf{M}_{ab}$ is the probability that the number of crimes of type *b* committed by perpetrators who committed both crimes of type *a* and *b* is smaller than $\textbf{M}_{ab}$, i.e.,

3 $$\begin{aligned} p_{val}(\textbf{M}_{ab}) = 1- \sum _{x= \textbf{N}_{ab}}^{\textbf{M}_{ab}-1} \frac{{{x-1} \atopwithdelims (){\textbf{N}_{ab}-1}} {{\textbf{M}_b - x-1} \atopwithdelims (){\textbf{N}_b - \textbf{N}_{ab}-1}}}{{{\textbf{M}_b-1} \atopwithdelims (){\textbf{N}_b-1}}} \,. \end{aligned}$$

Note that the summation index *x* goes from $\textbf{N}_{ab}$ since each perpetrator committed at least one crime of both types, *a*, and *b*. Again, the *p*-value *p* has to be corrected for multiple hypothesis testing, and we use the conservative Bonferroni correction. The statistically validated directed network is denoted as $\mathscr {M}_{ab}$.

For computational purposes, since factorials of large numbers are computationally demanding, it is convenient to express the hypergeometric distribution in Eq. (2) using the recursive formula

4 $$\begin{aligned} p(\textbf{M}_{ab}+1|\textbf{N}_{ab},\textbf{M}_b,\textbf{N}_b) = \frac{\textbf{M}_{ab}\, (\textbf{M}_b-\textbf{M}_{ab}-\textbf{N}_b+\textbf{N}_{ab})}{(\textbf{M}_{ab}-\textbf{N}_{ab}+1)\, (\textbf{M}_b-\textbf{M}_{ab}-1)}\, p(\textbf{M}_{ab}|\textbf{N}_{ab},\textbf{M}_b,\textbf{N}_b)\,. \end{aligned}$$

The probability of the lowest possible $\textbf{M}_{ab}=\textbf{N}_{ab}$, which reads

5 $$\begin{aligned} p(\textbf{N}_{ab}|\textbf{N}_{ab},\textbf{M}_b,\textbf{N}_b) = \frac{(\textbf{N}_b-1)!(\textbf{M}_b-\textbf{N}_{ab}-1)!}{(\textbf{M}_b-1)!(\textbf{N}_b-\textbf{N}_{ab}-1)!}\,, \end{aligned}$$

can be efficiently calculated by the method of decomposition of factorials into prime numbers^46^. The procedure is analogous to the one used for efficient calculation of original hypergeometric distributions^47^.

### Clustering of crime types

Given the statistically validated networks, we now apply community detection algorithms to derive a data-driven clustering of crime types according to their co-appearance within criminal careers. We extend the existing methodology by involving not only the number of crimes but also the number of perpetrators that commit both types of crime to define the statistically validated directed networks.

#### Community detection

To detect communities in the C-C network, we use the *Infomap* algorithm^48^ that is based on random walks on the network. One could equally employ other community detection methods such as the Louvain^49^ or Leiden method^50^. We use the Infomap algorithm, to be able to compare results directly with Tuminello et al.^32^, who used the same method.

The resulting community structure is displayed as a community-community network, where each node represents one community, $\alpha$. We denote the set of nodes as $\alpha ,\beta ,\dots$, where each community has its members (crime categories), e.g., $\alpha =\{c_1,\dots ,c_k\}$. The undirected community-community network is given by $\textbf{C}_{\alpha \beta } = \sum _{a \in \alpha , b \in \beta } \mathscr {N}_{ab}$. Thus the link weights correspond to the total number of perpetrators that committed crimes from both communities. We also define the directed links obtained from the statistically validated directed community-community networks as $\textbf{D}_{\alpha \beta } = \sum _{a \in \alpha ,b \in \beta } \mathscr {M}_{ab}$.

### Criminals’ trajectories and identifying level of specialization for communities of crimes

To define generalists and specialists we calculate a crime trajectory for each perpetrator. Each perpetrator is assigned a sequence of crime-type communities from the clustered crime-crime network. In particular, We denote a *trajectory* of a perpetrator *p* as $x^p_t\in \mathscr {C}$, which indicates that *p* committed crime $x^p_t$ at time *t*. By considering all perpetrators who committed more than one crime, we estimate the transition frequencies between the crime communities. To a first-order approximation, transitions between crime communities can be described as Markov chains with transition probabilities, $p(x_{t+1} \in \alpha |x_{t} \in \beta )$. By using the local mutual information^51^, $I_{\alpha \rightarrow \beta }$, between communities

6 $$\begin{aligned} I(\alpha \rightarrow \beta ) = \log _2 \frac{p(x_{t+1} \in \beta |x_t \in \alpha )}{ p(x_{t+1} \in \beta )} =\log _2 \frac{p( x_{t+1} \in \beta ,x_t \in \alpha )}{ p(x_{t+1} \in \beta )\cdot p(x_t \in \alpha )} \equiv \log _2 \frac{p(\alpha \rightarrow \beta )}{p(\beta ) \cdot p(\alpha )}\end{aligned}$$

we determine how often (compared to a random jump model) perpetrators jump from crime community $\alpha$ to crime community $\beta$. Assuming the distribution is stationary, one can omit the time index and denote the probability of observing crime from cluster $\alpha$ simply as $p(\alpha )$ and observing transition $\alpha \rightarrow \beta$ as $p(\alpha \rightarrow \beta )$. For the case where we observe no jump $\alpha \rightarrow \beta$, the mutual information is minus infinity. We let $I(\alpha \rightarrow \beta )$ be undefined in this case.

Calculating $I(\alpha \rightarrow \beta )$ allows us to compare the Markov chain model to a null model of random jumps according to the probability of committing a crime from community $\beta$ given by $p(x_{t+1} \in \beta )$. This local information is not symmetric in its arguments since the former crime class denotes the source community, and the latter class denotes the target community. In general, local mutual information can be both positive and negative. If $I(\alpha \rightarrow \beta ) >0$, the frequency of jumps between the groups is higher than expected from the null model; if $I(\alpha \rightarrow \beta ) <0$, the frequency is smaller. Particularly interesting is the local mutual information of transition within one community, i.e., $I(\alpha ):= I(\alpha \rightarrow \alpha )$, which measures the tendency of a perpetrator to remain in the community when committing two subsequent crimes. The value of $I(\alpha )$ means that $p(\alpha \rightarrow \alpha ) = \left( 2^{\frac{I(\alpha )}{2}} p(\alpha )\right) ^2$, so according to the null model of random jumps, the frequency of observing crimes from cluster $\alpha$ is rescaled by a factor $2^{\frac{I(\alpha )}{2}}$, so $I(\alpha )/2$ is the rescaling exponent determining the deviation from the null model. Intuitively, the mutual information quantifies how often the perpetrators commit two consecutive crime categories after each other compared to the situation where they commit the crimes randomly.

Particularly, the intra-community mutual information $I(\alpha )$ tells us how much more (or less) often a perpetrator commits two consecutive crimes in one crime cluster compared to the probability that two consecutive crimes committed will be from the same crime cluster. Intuitively, the mutual information measures how much more (or less) we observe that two consecutive crimes of one perpetrator will be from crime clusters $\alpha$ and $\beta$, compared to the situation when two independent perpetrators commit the two crimes. Particularly, the value $I(\alpha )$ tells us that one particular perpetrator commits two consecutive crimes from cluster $\alpha$ at least $2^{I(\alpha )/2}$ more often than two distinct randomly chosen perpetrators.

### Definition of specialists and generalists

We are now ready to use the obtained clustering of crime types to identify specialists and generalists. We define a specialist as any criminal charged with crimes from only 1 of the 21 identified clusters. Criminals charged with crimes from multiple clusters within their careers are considered generalists. In subsequent analyses of the differences between specialists and generalists, we focus on a subset of the data consisting of *repeat-offenders*. This consists of a subset of 64k individuals who were charged with at least five crimes in the dataset spanning six years. This selection criteria mitigates to some extent a limitation of our data: that we cannot distinguish whether individuals were charged with multiple crimes in a single event.

### Geographic range of criminal activity

Although most criminals tend to operate within a limited geographical range, some seem more effective when diversifying their activity locations^52^. We define the geographic range of an individual by calculating a quantity known as the *radius of gyration* on the locations of their criminal activities. Individuals committing crimes consistently in the same locations have low, more mobile ones –with activities in various regions– have a high radius of gyration.

For each offense, we geolocate the region’s latitude/longitude centroid, *r*. We generate a vector of offense locations for each perpetrator *i*: $\vec {r}_{i\mu } = (x_{i\mu }, y_{i\mu })$, at location index $\mu = 1... N_{locations}$, where *x* and *y* represent longitude and latitude, respectively. We calculate the average location as where the perpetrator is usually active, comparing the individual crime locations $\vec {r}_{i\mu }$ to the centroid of the criminal’s history $\overline{r}_i = \frac{\sum _\mu \vec {r}_{i\mu } }{\sum _\mu }$.

The *radius of gyration* $R_G$ is calculated as the square root of the mean of the squared distances, *d*, (calculated as the Haversine distance, which calculates a distance in meters from latitude and longitude coordinates given in degrees) of the locations $\vec {r}_{i\mu }$ to the individual’s centroid $\overline{r}_i$:

7 $$\begin{aligned} R_{\textrm{G},i} = \sqrt{\frac{\sum _\mu d(\overline{r}_i, \vec {r}_{i})^2}{\sum _\mu }} \,. \end{aligned}$$

### Collaboration network

To study the relationship between specialization and interactions between criminals, we derive a second network from the dataset that maps the collaboration between criminals. Specifically, nodes in this network are individual criminals connected by an edge if they collaborated on a specific crime event in our database. Edges have greater weight as criminals collaborate more often. Specifically, we define the collaboration network as a matrix ${\mathscr {C}}$. The entry ${\mathscr {C}}_{cd}$ quantifies the collaboration between criminals *c* and *d*. Specifically, ${\mathscr {C}}_{cd}=\sum _k \dfrac{\delta ^{k}_{c}\delta ^{k}_{d}}{n_{k}-1}$, where $\delta ^{k}_{c}$ is equal to 1 if criminal *c* participated in crime event *k*, $n_{k}$ is the number of criminals involved in crime event *k*, and the sum is over all crime events in the dataset.

This edge weighting method is sometimes called Newman’s hyperbolic weighting method^53^, in which the contribution of a specific criminal collaboration to the weight between two criminals is inversely proportional to the number of collaborators on that crime. For example, if two criminals collaborate on a specific crime alone as a pair, the edge between them will have a higher weight than that between two criminals who collaborate on a specific crime with ten other collaborators. We construct the collaboration network including all individuals charged with a crime in our dataset. In the analysis below, we contrast specialists’ and generalists’ characteristic positions and connectivity patterns in this collaboration network.

## Results

### Crime clusters

The 21 identified crime clusters, i.e., the nodes in the community-community network $\textbf{C}_{\alpha \beta }$, from the clustering of the statistically validated C-C network, $\mathscr {N}_{ab}$, are summarized in Table 1. Since crimes naturally cluster according to crime domains, we label the clusters by designations such as economic crimes, violent crimes, street criminality, etc. The table contains the number of crime types (paragraphs in the criminal code) that belong to the community, the number of crimes committed there, and the number of involved perpetrators. Several example crimes that belong to the community are mentioned in the rightmost column. Note the community names were chosen to be descriptive for the most crimes contained in the community; some crimes might not fully correspond to the community name.

**Figure 1:**
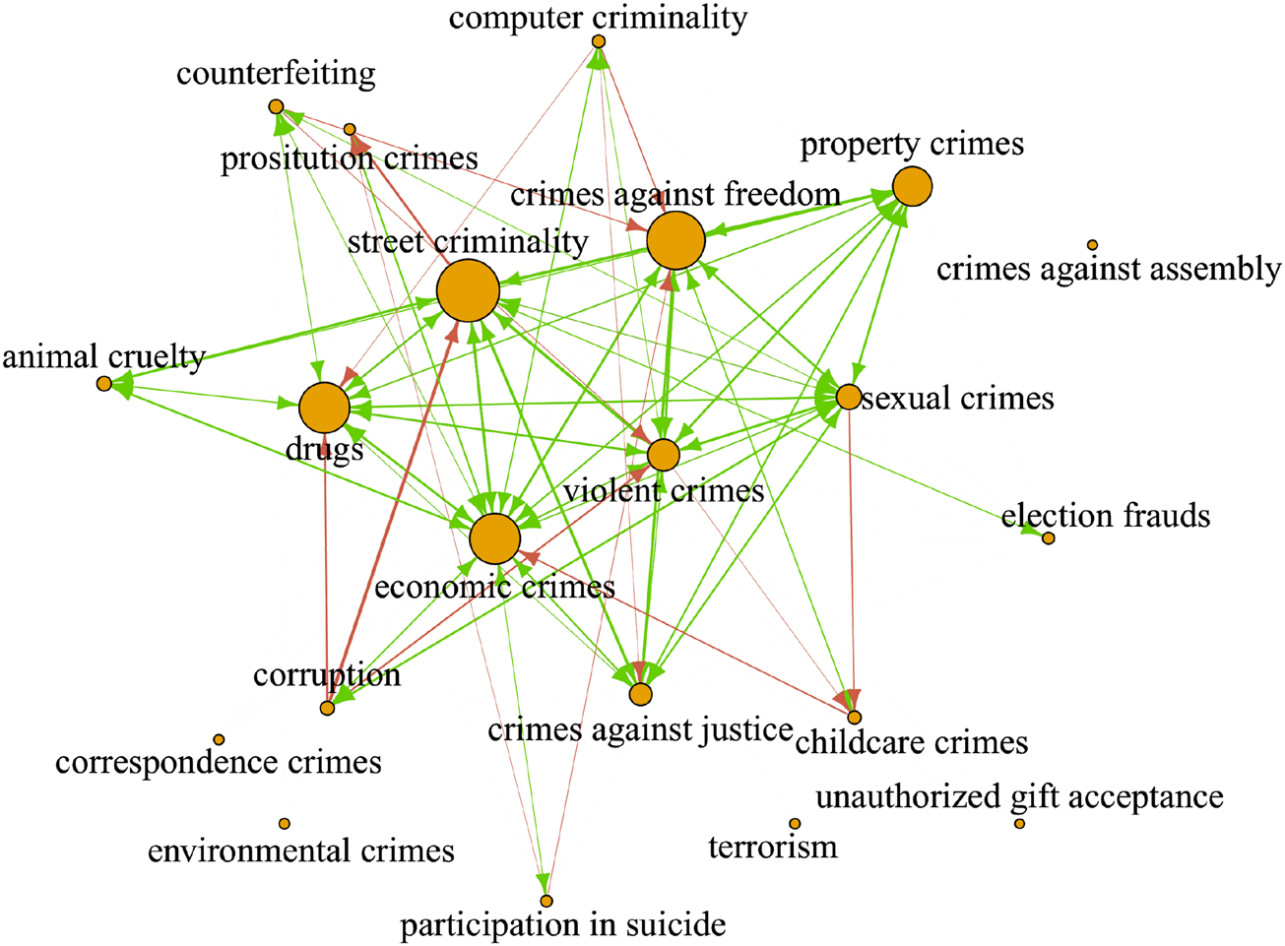
Transitions between crime clusters. Nodes represent the crime clusters; their size corresponds to the number of criminal acts. Green arrows indicate that all three link types, i.e., links between communities calculated from the undirected community-community network $\textbf{C}_{\alpha \beta }=\textbf{C}_{\beta \alpha }$, and directed links from the directed community-community network in both directions, i.e., $\textbf{D}_{\alpha \beta }$, and $\textbf{D}_{\beta \alpha }$, are all statistically significant. The link width represents the number of criminal acts committed by the perpetrators in both crime clusters, $\alpha$, and $\beta$. Green arrows are typically observed between large crime clusters e.g., ”street criminality”, ”crimes against freedom”, and ”violent crimes”. Red arrows represent statistically significant links in the undirected network, $\textbf{C}_{\alpha \beta }=\textbf{C}_{\beta \alpha }$, and a link of the directed (validated) network in one direction only (i.e., $\textbf{D}_{\alpha \beta }$ is significant but $\textbf{D}_{\beta \alpha }$ is not). These are typically observed between large to small crime clusters, as e.g., ”corruption” to ”street criminality”, ”prostitution” to *”crimes against freedom”*, or ”sexual crimes” to ”childcare crimes”.

The crime community-community network is depicted in Fig. 1. Nodes are the crime clusters; the size of the nodes corresponds to the number of crimes committed, and the link width represents the number of crimes committed by perpetrators that committed crimes in both clusters. The connection between a pair of crime clusters, say $\alpha$ and $\beta$, can be described by three numbers: the value of the undirected community-community network link $\textbf{C}_{\alpha \beta } = \textbf{C}_{\beta \alpha }$, and the two directed links of the directed community-community network, i.e., $\textbf{D}_{\alpha \beta }$ and $\textbf{D}_{\beta \alpha }$. Depending on whether the links are significant (with respect to the hypergeometric filtering), we can divide the links between communities into three categories.

First, the links where all $\textbf{C}_{\alpha \beta }$, $\textbf{D}_{\alpha \beta }$ and $\textbf{D}_{\beta \alpha }$ are statistically significant. These links show the strong relations between the clusters in both directions and are depicted in green in Fig. 1. A typical example of such a link is ”street criminality” and ”crimes against freedom”. The second type are those links where $\textbf{C}_{\alpha \beta }$ and $\textbf{D}_{\alpha \beta }$ are significant, but $\textbf{D}_{\beta \alpha }$ is not significant. These are most interesting since they enable us to reveal the structure of the link, as the link indicates that the perpetrators committing crimes from cluster $\alpha$ also commit crimes from cluster $\beta$ but not vice versa. These links are depicted in red in Fig. 1. Finally, the third case is when either $\textbf{D}_{\alpha \beta }$ or $\textbf{D}_{\beta \alpha }$ are significant but $\textbf{C}_{\alpha \beta }$ is not significant. These links are not depicted in the figure for the sake of clarity.

The list of observed crime communities and their properties is summarized in Table 1. We observe six large crime clusters, i.e., economic crimes, crimes against freedom, street criminality, drug crimes, violent crimes, and property crimes. These clusters appear in the center of the community network; the strongest connections are between street crimes, violent crimes, and crimes against freedom, constituting a strong triangle. Crimes against freedom have a strong link to property crimes. Similarly, there are strong links between street criminality, drug crimes, and economic crimes. All links are bi-directional, and these six clusters are strongly connected. The remaining clusters are connected only to a few other clusters; some of the links are uni-directional and typically go from smaller to larger communities. We mention a few examples: (a) a link from computer criminality to drug crimes, which might point to online drug sales, (b) links from sexual crimes and violent crimes to childcare crimes, which corresponds to child sexual abuse, and violence against children, respectively, (c) links from corruption to drugs crimes and street criminality, pointing to the fact that corruption is often connected with other criminality where criminals try to bribe police officers or witnesses, (d) a link from street criminality to prostitution and, consequently, a link from prostitution to crimes against freedom and participation in suicide.

**Table 1:** Characterization of crime clusters of the statistically validated crime network.

Community name	# crime types	# acts	# perpetrators	Main crime types
Economic crimes	54	238,221	160,772	Fraud, embezzlement, forgery,
				Document falsification, money laundering,
				Faked bankruptcy, use of foreign documents,
				Illegal gambling, pyramid games,
				Criminal organization, usury
Crimes against freedom	15	331,138	254,165	Coercion, threat, battery, blackmailing,
				Forced marriage, kidnapping, slave trade
Street criminality	17	394,491	199,929	Theft, suppression of documents
				Unauthorized use of vehicles, use of fake money,
				Hazardous handling of explosives, defalcation
Drug crimes	15	236,980	150,300	Drug possession, drug trafficking
				Illegal energy consumption
Violent crimes	20	73,241	63,771	Bodily injury, murder, robbery, mayhem
				Abandoning an injured person
				Assault of a police officer, breach of the peace
Sexual crimes	24	36,701	31,687	Rape, sexual harassment, incitement
				Illegal spreading of pornography,
				Sexual abuse of children, incest
Property crimes	13	127,340	84,318	Property damage, trespassing, arson
				Confiscation of property
				Transfer of border marks
Crimes against justice	11	25,372	23,531	Perjury, insurance fraud,
				Defamation, criminal facilitation
Corruption	12	3,612	3,229	Bribery, abuse of power, benefit allowance
Computer criminality	11	2,170	2,008	Misuse of data (private/banking/corporate)
				Misuse of computer programs
Prostitution crimes	6	923	836	Pimping, international prostitution trade
				Human trafficking
Animal cruelty	5	4,592	4,175	Animal cruelty
				Intervention of hunting or fishing law
Election frauds	6	1,321	1,094	Election falsification
Childcare crimes	5	2,732	2,603	Child neglect, child abduction
Terrorism	4	343	324	Terrorism financing, terrorism approval
Counterfeiting	3	4,005	3,080	Counterfeiting
Participation in suicide	4	1,194	1,185	Facilitating suicide
Environmental crimes	3	431	378	Intentional damage to the environment
Crimes against assembly	2	95	95	Preventing/disturbing an assembly
Unauthorized gift acceptance	2	47	45	Acceptance of gifts by authorities
Correspondence crimes	1	261	239	Violation of the secrecy of correspondence
Total	233	1,485,641	987,764	Unique perpetrators: 581,486

Each community is characterized by its crime composition, number of crime types (corresponding to the specific paragraph of the criminal code), number of acts, and number of perpetrators. The rightmost column provides several examples of representative crimes within every crime cluster. Note that the number of unique perpetrators is not the sum of the perpetrators in each cluster since some of the perpetrators were committing crimes in several crime clusters.

### Criminal trajectories and level of specialization of criminal communities

**Figure 2:**
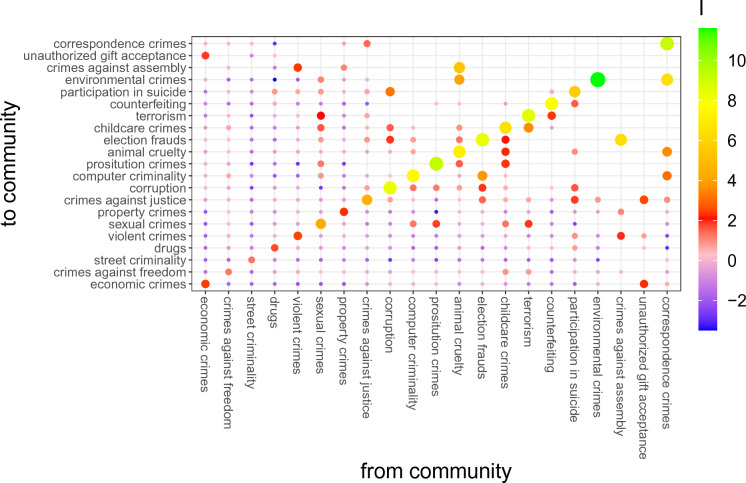
Local mutual information $I(\alpha \rightarrow \beta )$ between crime communities. It indicates how much more (less) often a perpetrator commits two consecutive crimes from crime clusters $\alpha$ and $\beta$, compared to the frequency of committing crimes from crime clusters $\alpha$ and $\beta$. The local mutual information is encoded both by color (see the color scale) and by size (for positive *I*, the size of the point is proportional to *I*.) High (orange to green) values on the diagonal highlight those crime types that criminals tend to stay within, suggesting specialization.

We calculate the local mutual information, $I(\alpha \rightarrow \beta )$, for the 21 crime clusters. Here we use a reduced dataset, where from the total of 581k different perpetrators in the data, we look at the subset of 131*k* who committed more than one crime. Results are depicted in Fig. 2. The local mutual information is encoded both by color (see color-scale) and size (for positive *I*, the larger the local mutual information, the larger the dot). A special role is played by the diagonal of the matrix, i.e., $I(\alpha \rightarrow \alpha ) = I(\alpha )$ that represents the relative frequency of continuing in the criminality of the same type. Individuals who stay within the same type were identified as *specialists*. We observe that $I(\alpha ) >0$ for all communities, which means that committing two subsequent crimes in the same cluster is more probable than in the null model, which is in agreement with the crime clusters from the previous section.

The value of $I(\alpha )$ changes considerably between different communities. This allows us to associate crime clusters with several types. Remember that $I(\alpha )/2$ represents the characteristic rescaling exponent of a crime cluster, $\alpha$. To obtain a threshold for the distinction between crime clusters of crimes committed by generalists and specialists, we choose $I_{crit}= 4$, so $2^{(I_{crit}/2)} =4$, which means that for specialists we have $p(\alpha \rightarrow \alpha ) \ge (4 p(\alpha ))^2$. So the probability that a perpetrator commits two consecutive crimes from one crime cluster $\alpha$ is at least 16 times as high as the probability that the two crimes are committed by two random perpetrators.

We obtain that crime clusters with $$I(\alpha )<I_{crit}$$ are: economic crimes ($$I=2.42)$$, crimes against freedom ($$I=1.27$$), street criminality ($$I=1.31$$), drug-related crimes ($$I=1.73$$), violent crimes ($$I=2.50$$), and property crimes ($$I=2.29$$). These crimes are, therefore, typically committed by generalists. This analysis confirms previous findings that there is little tendency for individuals to specialize in violence^33^.

On the other hand, crimes with $$I(\alpha ) > I_ {crit}$$ include sexual crimes ($$I=4.27$$), crimes against justice ($$I=4.49)$$, corruption ($$I=8.32$$), computer criminality ($$I=7.60$$), prostitution ($$I=9.30$$), animal cruelty ($$I=7.13$$), election frauds ($$I=8.39$$), childcare crimes ($$I=6.57$$), terrorism ($$I=8.47$$), counterfeiting ($$I=7.91$$), participation in suicide ($$I=5.66$$), and environmental crimes ($$I=11.59$$). Finally, the remaining crime communities, i.e., crimes against assembly, unauthorized gift acceptance, and correspondence crimes, are so rare that the number of transitions is too small to make a valid classification.

Further, we observe that in several cases, the local information is significantly positive between different clusters, i.e., $I(\alpha \rightarrow \beta ) \ge 2$, where $\alpha \ne \beta$. For example, we observe significant local levels of mutual information between economic crimes and unauthorized gift acceptance. Moreover, we observe that prostitution crimes and sexual crimes also have significant local mutual information, which is plausible due to the common sexual nature of both clusters. Most interesting are the cases when $I(\alpha \rightarrow \beta )$ is much higher than $I(\beta \rightarrow \alpha )$. To these significantly asymmetric transitions belong: election frauds $\rightarrow$ computer criminality, animal cruelty $\rightarrow$ environmental crimes and crimes against assembly, crimes against assembly $\rightarrow$ election frauds, and corruption $\rightarrow$ participation in suicide. An interesting aspect here is that some of the links are not significant when compared with the links between crime clusters shown in Fig. 1.

### Characteristics of specialists versus generalists

Given the crime clusters, we classify individual perpetrators as generalists or specialists according to our definition: specialists are those individuals staying within a single crime cluster across their careers. From all 581*k* different perpetrators, we take the reduced set of those individuals charged five times in our data (called repeat offenders). This excludes low-frequency offenders who could have been mislabeled as specialists. Among the 64*k* repeat offenders, we categorize 11*k* (17%) individuals as specialists and 53*k* (83%) as generalists.

The average number of crimes committed by specialists and generalists is roughly equal (11.22 for generalists vs 11.26 for specialists). Generalists commit a median of 8 crimes vs a median of 7 for specialists. This observation is in line with previous work that differences in offending frequency between specialists and generalists are minimal^54^. On the other hand, as we have only six years of data, any hypothesized tendency of specialists to become generalists over time may not be visible in our dataset^33,55^.

#### Socio-demographic differences of specialists versus generalists

We connect other socio-demographic information to criminals and provide statistical evidence of over- and under-representation of specific traits in the two respective populations. Women are significantly more likely to be specialized than men (26% of women vs. 16% of men, $p<0.01$ Mann–Whitney U). Individuals under the age of 20 are highly versatile, with a specialist rate of only 11%, vs. 13% for those between the age of 20 and 30. 21% of individuals older than 30 were defined as specialists based on their activity across six years of data. These findings align with previous empirical findings from the literature carried out at smaller scales, which we revisit in the discussion.

#### Mobility of specialists versus generalists

**Figure 3:**
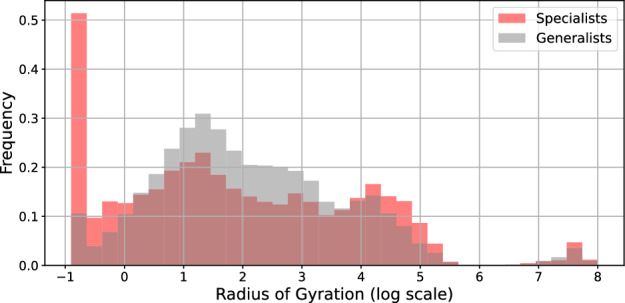
Distribution of the radius of gyration of repeat-offender specialists (red) and generalists, on a logarithmic scale. We see that the majority of specialists are charged with crimes in the same place and –if they move– they tend to move slightly larger distances. Generalists, on average, show greater mobility.

Specialization has a significant correlation with the geographic range of action of individual perpetrators. Figure 3 shows the distribution of the (log) radius of gyration, *R*, for specialist (red) and generalist (gray) repeat offenders. We observe that specialists are far more likely to commit crimes in the same place and that generalists tend to be more mobile.

To exclude the possibility that the observed difference is a statistical artifact from the situation that if generalists are more active (i.e. commit more crimes), they may –by chance– commit crimes in a greater variety of locations we test for the statistical significance of this difference while controlling for overall activity. Indeed, past work debates whether the frequency of offending may be a confounding factor in the observation of specialization^33,54^. We fit a linear regression model predicting a criminal *i*’s (log) radius of gyration of the form

8 $$\begin{aligned} \log R_{\textrm{G},i} = \beta _{0} + \beta _{1} S_{i} + \beta _{2} \log N_{i} + \varepsilon \,, \end{aligned}$$

where $S_{i}$ is a binary variable that is 1 if the criminal *i* is a specialist and 0 if he is a generalists. Here $N_{i}$ is the number of crimes in the observed career of a criminal, *i*, $\beta _{0}$ is an intercept, and $\epsilon$ is the error term. Results are in Table 2. Specialists tend to operate in a much more geographically confined area. Controlling for how many crimes they commit, specialists’ radius of gyration is, on average, 19% lower than that of generalists.

**Table 2:** Linear regression (OLS) results predicting individuals’ criminal log radius of gyration. We report the estimated coefficients of equation 8, their standard errors, *t*-statistic, and the resulting *p*-values.

	Coefficient	Std. err.	t-statistic	*p*-value
Intercept ($\beta _0$)	1.15	0.025	45.7	$<0.001$
Specialist ($\beta _1$)	− 0.19	0.017	− 11.3	$<0.001$
log(# Crimes) ($\beta _2$)	0.45	0.011	40.5	$<0.001$
Observations	64406		Adj. $R^{2}$	0.027

Controlling for the number of crimes, specialists have a radius of gyration of around 19% lower than generalists on average.

### Position of specialists and generalists in the collaboration network

We report summary statistics about the positions of specialists and generalists in the collaboration network, ${\mathscr {C}}$, described in the methods in Table 3. For each criminal with at least five offenses in the last five years, we derive the following network characteristics:

- *Degree*: number of collaborators.
- *Strength*: number of collaborations, counting repeated collaborations with others.
- *2-step neighbors*: number of criminals within two steps of a criminal.
- *clustering coefficient*: shared pairs of neighbors of the criminal that are connected themselves.
- *Has-network-connection*: if a criminal has any connection at all.
- *Strength/degree*: ratio of strength to degree of the criminal.

We study the ego networks of individuals in our dataset. Ego networks consist of the collaborators of a focal node, the “ego”, and the connections between them. We find that generalists tend to have larger local networks than specialists: they have more direct connections (degree) (mean 2.98 vs 2.11, Mann–Whitney U *p*-value $<0.01$). Beyond the ego-network level, generalists also have more two-step neighbors (mean 5.27 vs 11.74, Mann–Whitney U *p*-value $<0.01$). Generalists are slightly more likely to have any collaborations at all than specialists (58% vs 66% - Mann–Whitney U *p*-value $<0.01$). On the other hand, specialists have more repeated connections (average strength 5.49 vs 3.26; a higher strength to degree 1.57 vs 0.66; both have a significant Mann–Whitney U *p*-value of $<0.01$). Specialists tend to have slightly more closed ego networks, as seen in the average clustering coefficient of 0.27 vs 0.23 for generalists (significant difference at $p<0.01$).

**Figure 4:**
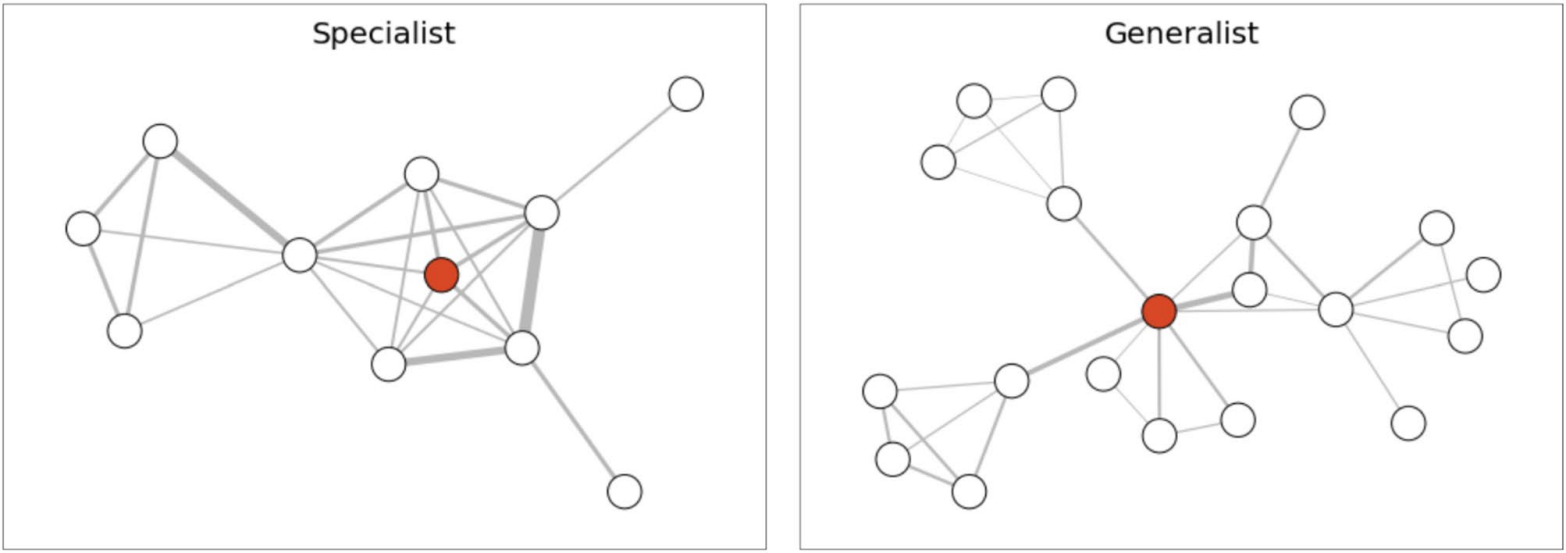
Characteristic collaboration networks of specialists and generalists. The two-step ego networks of a specialist and generalist criminal (red nodes) highlight characteristic differences in their collaboration networks. Generalists have larger, more open networks, while specialists have smaller, more closed networks characterized by repeated collaborations.

Figure 4 shows two characteristic two-step ego networks for specialists and generalists, providing a visual representation of the stylized patterns observed in Table 3. The ego node is highlighted in red; we include all alters up to two steps away, as well as the connections between them. For instance, the specialist node is embedded in a clique: all five of their direct connections are themselves connected with each other. The thicker edges, apparent in the specialist’s extended network, highlight repeated collaborations. The generalist’s network, on the other hand, has significantly lower clustering. While the generalist has a higher degree (7 direct connections), there are fewer repeated connections and hardly any interactions among his direct neighbors themselves.

**Table 3:** Specialists and generalists network position summary statistics..

	Degree	Strength	2-Step Neighbors	Clustering Coeff.	Has Network Connection	Strength/Degree
Specialists						
Mean	2.11	5.49	5.27	0.27	0.58	1.57
Std	5.41	16.04	11.84	0.42	0.49	5.44
Min	0	0	1	0	0	0
25%	0	0	1	0	0	0
50%	1	1	2	0	1	0.50
75%	2	6	5	0.60	1	1.75
Max	162	731	222	1	1	352
Generalists						
Mean	2.98	3.26	11.74	0.23	0.66	0.66
Std	5.08	9.67	23.35	0.35	0.47	1.85
Min	0	0	1	0	0	0
25%	0	0	1	0	0	0
50%	1	1	3	0	1	0.50
75%	4	3	11	0.36	1	0.70
Max	117	581	404	1	1	118.25

## Discussion

In this work, we presented an empirical study of specialization in a large dataset of criminal behavior. Motivated by the observation that defining specialization using legal code sections can lead to artificial groupings we developed a method to cluster frequently co-occurring crime types within individual careers. The resulting clustering provides a data-defined categorization of crimes in which patterns of offending behavior can be observed. We use it to define specialists in the population of criminals as those who stay within one category throughout their careers.

Applying this method to a comprehensive dataset of criminal activity of a whole country over six years, we demonstrate how the method can be used to cluster crime types and define specialization. We use the results to study the socio-demographic and mobility characteristics of specialists and generalists. We can further locate specialists and generalists within their criminal collaboration networks and interpret these positions. Our work is part of an emerging field that applies the methods of data and network science^56–58^ to study criminal behavior^29,59–61^.

Our method to cluster crime types extends a statistical method from Tumminello et al.^32^. Adapted to our data, we recover 21 crime clusters. Using the simple information-theoretic concept of mutual information, we show that transitions are less likely out of certain crime types, suggesting that specialization is much more likely in certain categories than others^33^. A strength of the new method is that each cluster can consist of a different number of crime types and offenses. Some crimes (such as fraud or drug possession) are much more common than others (counterfeiting or misuse of data). The method also extends the previous state of the art by considering how often individuals commit specific kinds of crimes when generating clusters. Observed clusters of types of criminal activity do not neatly match with categorizations in the legal code, suggesting that a data-driven clustering like the one we carry out may be a more appropriate way to define specialization. Future work should investigate the comparability of classifications of criminal behavior based on legal codes in different jurisdictions with those derived from data on criminal behavior, perhaps using international categorization like the ICCS as a basis for comparison^40^.

Our main empirical contribution is the presentation of large-scale evidence on socio-demographic and behavioral differences between specialist and generalist individuals. We found differences by gender and age in line with most previous work. We observe specialization among older individuals in our dataset^2,5^. As our data covers six years, we cannot tell whether early or late-onset criminal careers are predictive of future specialization or versatility^2^. Women are more likely to be specialists than men as observed in a variety of alternative settings^18,32,62^.

On the other hand, previous work on the geographic range of specialists versus generalists is much more limited, likely owing to a lack of comprehensive data covering a larger geographic area. One work on organized criminal groups suggests that localized activity tends to be generalist^21^. In the case of our dataset that covers all crimes and not just organized crime, we find evidence that it is rather specialists that tend to stay in the same place. Indeed, controlling for the number of crimes committed, we find that specialists have a 19% lower radius of gyration than comparable generalists. That specialists tend to concentrate their activity in a specific region suggests that they rely on knowledge of a place and perhaps the support of individuals in a specific area to be effective. Criminologists have long understood criminal mobility in terms of opportunity: travel to a new place is costly and full of uncertainty^63,64^. Our findings suggest that these costs are higher for specialized criminals. In other words, specialized criminal behavior may benefit from knowledge about a specific place or from repeated collaboration which is more easily coordinated in a small geographic area. One potential extension of our work would be to relate observed criminal mobility to the clustering of crimes in space^65^.

A further contribution is our finding that specialists and generalists have different collaboration patterns, measured via their position in collaboration networks. As in the case of geographic differences, collaboration differences between specialists and generalists are not widely studied in the literature on criminal careers. Specialists have smaller but denser, more tightly-knit collaboration networks. They are more likely to collaborate repeatedly with the same partners. This suggests that specialists are more effective when collaborating closely with others. These intensive interactions may be important channels for learning and dependency among specialists. For example, a high-level drug dealer may rely on others to launder profits. These, in turn, represent how an individual’s environment affects their inherent propensity to offend^8,16^. Previous work on specialization and collaboration among criminals has focused largely on organized crime as such organizations often exhibit a hierarchy of authority and a division of labor based on specialization and roles^21,66,67^. Indeed, specialization (and the skills developed by specialists) and collaboration play complementary roles functioning of criminal networks^24,25^.

In the general population of offenders, our results on collaboration network differences suggest that specialists are embedded in smaller collaborative environments characterized by repeated interactions. Interpreted through the lens of theoretical frameworks like state dependence or SAT, this suggests that specialists are embedded in networks that may be self-reinforcing^68^. For instance, repeated interactions with specialized criminals may indicate dependencies, whether they are social, psychological, or economic. Differences in such “social capital” of specialist and generalist offenders certainly merit future attention by the researchers of criminal careers.

Our study has several limitations. As with nearly all empirical studies of criminal behavior, our data likely suffers from selection bias. In other words, our data does not contain information about undetected or unsolved cases of criminality. This is a common limitation in data-driven studies of criminal behavior, which by nature are limited to prosecuted or highly visible activities^69^. At the same time, events in our data are when individuals are charged with crimes - not all events are correctly assigned to the guilty individual. Peculiarities of the legal system of the country from which our data are sourced may also influence the results.

Second, although we have several years of activity, criminal careers can span decades. Indeed, we can only observe specialization in a relatively brief part of any individual’s total life and potential criminal career. In this way, our analysis cannot reveal trajectories that lead to specialization. Data covering longer time periods could lead to deeper insights into individual paths through the world of crime.

Our data is not linked to information on incarceration. Certainly, a longer period of time in jail or prison would limit an individual’s ability to re-offend within our dataset. Future work should consider the consequences of incarceration itself on specialization, especially as an opportunity for the transfer of human capital or strengthening of social capital between criminals^70^. At the other end of the temporal scale, we also cannot tell whether specific charges very close to one another in time refer to single events or separate ones.

Despite these limitations, we claim our work presents novel and confirmatory results about the specialization in criminal careers. We do so by presenting a refined and generally applicable method to define and measure specialization at the scale of a whole country.

## Data Availability

The datasets generated and/or analyzed during the current study are not publicly available due to the confidentiality of the data as agreed with the project partner—Austrian Federal Criminal Police Office Department II/BK/4 - Criminal Analysis. Access to the data via the project partner can be arranged via the corresponding author on reasonable request.
